# Development and assessment of learning objects about intramuscular medication administration

**DOI:** 10.1590/0104-1169.3647.2472

**Published:** 2014

**Authors:** Lilian Mayumi Chinen Tamashiro, Heloisa Helena Ciqueto Peres

**Affiliations:** 1 Undergraduate student in Nursing, Escola de Enfermagem, Universidade de São Paulo, São Paulo, SP, Brazil. Scholarship holder of the Scientific Initiation Program at the Conselho Nacional de Desenvolvimento Científico e Tecnológico (CNPq), Brazil; 2 PhD, Full Professor, Escola de Enfermagem, Universidade de São Paulo, São Paulo, SP, Brazil

**Keywords:** Nursing, Nursing Informatics, Injections, Intramuscular

## Abstract

**OBJECTIVES:**

to develop and assess a learning object about intramuscular medication administration for nursing undergraduates and nurses.

**METHOD:**

a random, intentional and non-probabilistic sample was selected of nurses from a Brazilian social network of nursing and students from the Undergraduate Program at the University of São Paulo School of Nursing to serve as research subjects and assess the object.

**RESULTS:**

the participants, 8 nurses and 8 students, studied the object and answered an assessment instrument that included the following criteria: educational aspects (relevance of the theme, objectives and texts/hypertexts), interface of the environment (navigation, accessibility and screen design) and didactic resources (interactivity and presentation of resources). In total, 128 significant answers were obtained, 124 (97%) of which were positive, assessed as excellent and satisfactory, considered as a flexible, dynamic, objective resources that is appropriate to the nursing learning process.

**CONCLUSION:**

the educational technology shows a clear and easily understandable language and the teaching method could be applied in other themes, contributing to the education and training of nursing professionals, positively affecting nursing teaching, stimulating the knowledge, autonomous and independent learning, aligned with the new professional education requirements.

## Introduction

In nursing, many people consider the theme intramuscular medication administration (IM) a relatively simple procedure. Nevertheless, both professional practice and the literature review present various reports of complications related to this procedure. In the literature, reports were found of severe errors the nursing team committed in performing this procedure, which resulted in the formation of an abscess, local pain or in the limb where the medication was applied, reduced limb sensitivity, local necrosis, skin and fatty tissue atrophy, shoulder movement contraction and limitation, hematoma, nodulation, erythema, among others^(^ 1 ^)^.

Hence, it is considered that changing this reality is related to nursing staff education and training, aiming to guarantee the accomplishment of safe practices for the patient^(^ 1 ^)^. When discussing the learning process and its new trends, some considerations about the method applied in teaching processes at Brazilian colleges are relevant, mainly regarding new teaching technologies focused on the autonomy and independence of college students.

Reflecting on the current Brazilian educational system is important in view of the urgent need for reformulation, mainly in the development of didactical materials to adapt to the new professional education requirements deriving from Information and Communication Technologies (ICTs), breaking with the idea of isolated science, strengthening its trends towards interdisciplinarity, increasingly driving people towards its use in different knowledge areas^(^ 2 ^)^.

The use of ICT in Nursing teaching can grant interactive, dynamic, attractive and multisensorial experiences, supporting the improvement of the teaching-learning process^(^ 3 ^)^. In that context, the learning objects (LO) can be highlighted, characterized by flexible learning environments, compatible with active learning methods that value the students' autonomy^(^ 4 ^)^.

The Los can easily be reused in different learning contexts, reducing the costs of purchasing programs and installation licenses, besides the capacity to be used in any teaching platform around the world, offering the advantage of practicality for the sake of fast and safe updates.

In that perspective, the development of this object about intramuscular medication administration can positively influence nursing teaching, stimulating the knowledge and autonomous and independent learning, aligned with the new professional education requirements.

Based on this context, the objectives in this study were to: develop the learning object about intramuscular medication administration and assess it from the perspective of nursing undergraduates and nurses, with a view to testing this complementary teaching method, verifying the possible aspects, whether positive or negative, and proving the hypothesis about its probable positive impact in nursing teaching.

## Method

This exploratory and descriptive study is characterized as an applied technological production research, undertaken between 2012 and 2013. The study context was the Telenursing Center of the University of São Paulo School of Nursing (CETEnf - EEUSP), which is a virtual laboratory that offers support for the creation of pedagogical products, serving as an educative space for theoretical-practical teaching and research development in telenursing and nursing informatics^(^ 5 ^)^.

The creation of the LO followed the cyclical phases of analysis, design, development, implementation and assessment, proposed by Filatro^(^ 5 ^)^. In the *Analysis *phase, needs for the implementation of learning objects are surveyed. The *Design *involves the selection of pedagogical and technological strategies, as well as the description of the educational objectives. The *Development *comprises the production and adaptation of digital materials, assemblage and configuration of environments. The *Implementation *phase constitutes the didactical situation itself, when the instructional design proposal is applied. The *Assessment *includes the evaluation of the educational aspects, environment interface and didactical resources by the students and nurses.

The population sample was random, intentional and non-probabilistic. An invitation letter to participate in the study was sent to the target public through a message posted in a social network *(*Facebook), directed at the Brazilian community of Nurses and groups on this same network, constituted by students from EEUSP and the University of São Paulo at Ribeirão Preto College of Nursing. The letter presented the proposed LO and asked anyone interested to return the message by e-mail, requesting the forwarding of the Free and Informed Consent Form. After they had signed it, the tutorial to register in Moodle and access the LO were made available.

Only 156 people manifested their interest in participating in the study and only 16 (8 undergraduates from EEUSP and 8 nurses) answered the assessment instrument of the LO, due to the available time. Initially, a 20-day deadline was set to return the assessment form and, as a result of the small demand within that deadline, it was extended to another 30 days. According to the NBR ISO/IEC 14598-6^(^ 6 ^)^, with a view to consistent results, software should be assessed by at least eight participants for each assessment category.

The assessment instrument was based on other studies^(^ 7 ^-^ 8 ^)^ and primarily identifies the participants' profile, addressing data like education and length of professional experience in the area. Next, the assessment is presented of the criteria related to the educational aspects (relevance of the theme, objectives and texts/hypertexts), environment interface (navigation, accessibility and screen design) and didactical resources (interactivity and presentation of resources). Each criterion could be assessed through four numerical levels, represented as: 1 - Unsatisfactory; 2 - Reasonable; 3 - Satisfactory; 4 - Excellent. Finally, room was reserved at the end of the assessment instrument to describe the participants' possible comments and suggestions to improve the LO.

The data collected on the participants' assessment were registered in the form of absolute figures and organized in graphs according to the items assessed. The percentages of all characteristics assessed were related to the expected percentage of more than 70% of positive answers in order to be considered an appropriate LO. The data analysis was based on the theoretical framework about the study theme.

Approval for the research project was obtained from the Research Ethics Committee at the University of São Paulo School of Nursing (CEP/EEUSP), registered under process 1062/2011/CEP-EEUSP - SISNEP CAAE: 0068.0.196.000-11, on August 29^th^ 2011.

## Results

### Development of the Learning Object

**Figure 1:**
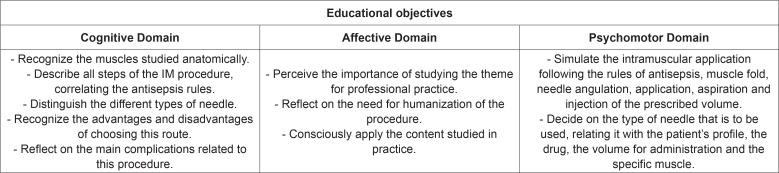
- Educational objectives of the learning object about intramuscular medication application, São Paulo, SP, Brazil, 2013

In the *analysis phase, *a bibliographic review on intramuscular medication administration was undertaken to identify learning needs and define the educational objectives of the LO. The articles analyzed showed that the nursing team commits severe errors in this procedure, such as the formation of abscess, local pain or in the limb where the medication was applied, reduced limb sensitivity, local necrosis, skin and fatty tissue atrophy, shoulder movement contraction and limitation, hematoma, nodulation, erythema, among others^(^ 1 ^)^. Based on the bibliographic reference framework, the educational objectives were elaborated according to Bloom's taxonomy^(^ 9 ^)^ in three domains: cognitive, affective and psychomotor, as shown in Figure 1.

In the *design phase*, storyboards were created to specify the LO in detail. The presentation editor *Microsoft* *(r)* *PowerPoint* *(r)* was used to create scenarios, texts, images, figures, the characters' dialogues and the sequence of the end product. In the *development phase*, the LO was actually constructed in the software *Articulate Storyline* *(r)*. This program contains tools with interactive resources that help to dynamically construct involving contents, including simulations, screen recordings, drag-and-drop interactions, clicking and revealing the activities, tests, evaluations, among other resources^(^ 10 ^)^. In the *implementation phase*, the LO was hosted on the *Moodle* platform, executed in a *Virtual Learning Environment *(VLE) of the USP student network.

**Figure 2:**
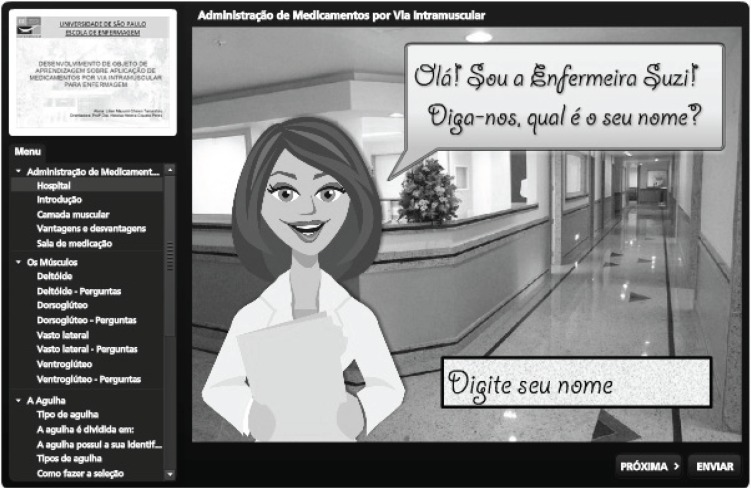
- Initial Screen of the Learning Object (LO) Intramuscular Medication Administration (IM), São Paulo, SP, Brazil, 2013

On the initial screen of the LO, a brief interaction with the user is presented through an avatar of a nurse, who serves as the main character in a hospital environment and invites the user to complete his/her name in the indicated field (Figure 2). Next, the objectives and themes are presented, as well as orientations for the interactions the user is supposed to accomplish during the study.

The themes addressed in the LO were: advantages and disadvantages of this route; identification and anatomy of the muscles: deltoid, dorsogluteal, vastus lateralis, ventrogluteal; presentation of different needle types and sizes; demonstration of asepsis technique and of IM procedure and main complications.

**Figure 3:**
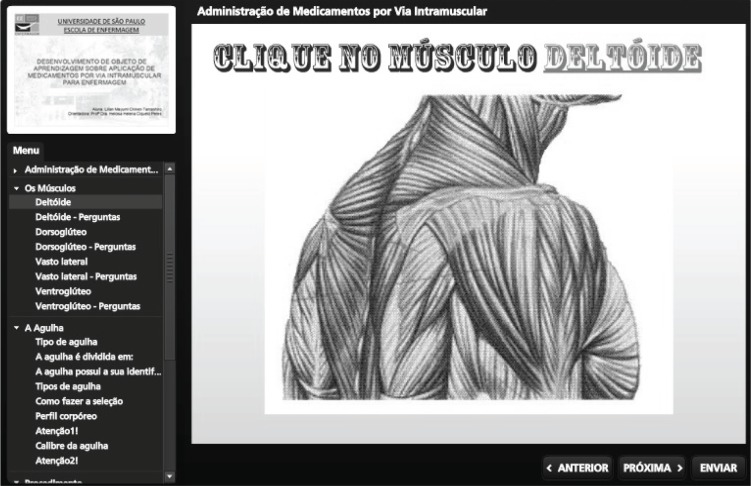
- Image of the interactive exercise about the anatomic muscle structure of the Learning Object (LO) Intramuscular Medication Administration (IM), São Paulo, SP, Brazil, 2013

Besides the texts and hypertexts described in the LO, interactive exercises were developed (Figure 3), a video about the medication administration preparation procedure, four three-dimensional videos with the anatomic structures of the muscles addressed, as well as a text file in the form of a checklist about the procedure steps.

The three-dimensional anatomic structures were developed through a Scientific Academic Cooperation Agreement between the subject Telemedicine at the University of São Paulo School of Medicine and the CETEnf-EEUSP, involving researchers, specialists and technicians responsible for the creation of the graphical resources. In the *evaluation phase, *faults were identified in the functioning and access to the LO and VLE and an orthographic review was undertaken.

### Evaluation of the Learning Object

**Figure 4:**
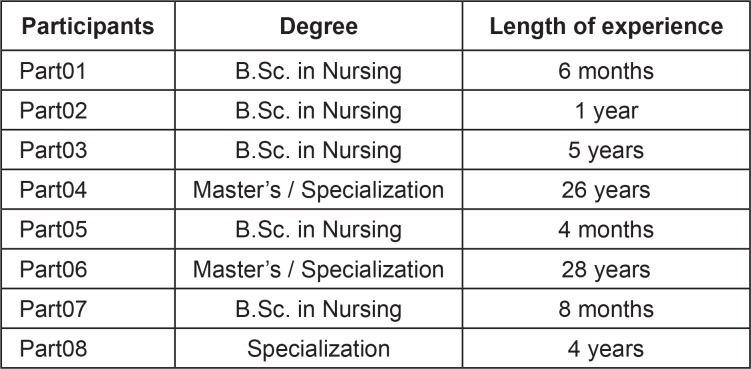
- Profile of the nurses. São Paulo, SP, Brazil, 2013

In the target public sample, 156 people manifested their interest in studying and assessing the LO, but only 16 (8 EEUSP undergraduates and 8 nurses) answered the assessment instrument. The nurses' profile was identified according to the degree and the length of experience, as demonstrated in Figure 4.

**Figure 5:**
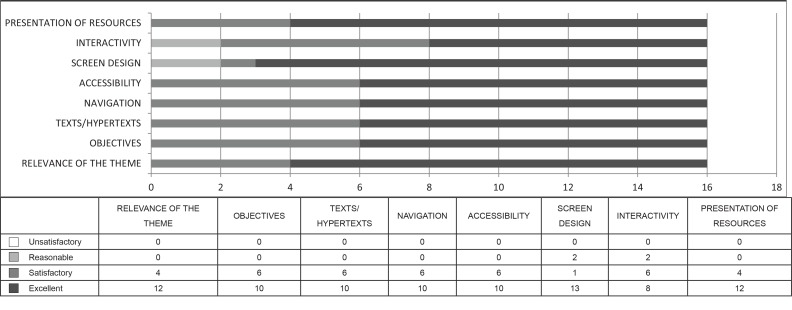
- Assessment of the Learning Object (LO) according to the participants' answers. São Paulo, SP, Brazil, 2013

In total, 128 significant answers were obtained to assess the LO, which was generally evaluated as excellent and satisfactory by the 8 nurses and 8 undergraduates, considering all assessment criteria, resulting in a total number of 124 (97%) positive answers. Therefore, it was considered appropriate for nursing teaching. These data are demonstrated in Figure 5.

As regards the evaluators' comments and suggestions for the LO, the following could be highlighted:

### Positive aspects

*(...) the language and audiovisual resources are very clear, easy to understand and complete* (Part16); *(...) I believe it will greatly contribute to the education of future Nursing professionals, because it is illustrative, clear and objective* (Part14); *(...) the interactive mode is a stimulating form of learning, all the more about this theme which is very important in professional practice* (Part10); *(...) the interactivity and balanced content also made the course very pleasant* (Part13); *(...) the theme is relevant, indicated since professional Nursing education, from the secondary to the higher level, also including Continuing Education, focused on health professionals* (Part06).

These reports demonstrate that the participants accepted the technology, suggesting the more frequent use of this method at educational institutions, contributing to the nursing teaching-learning process.

The texts serve as theoretical support tools for the users of the learning object, observing simplicity, clarity and objectivity. In combination with the animations, these are valuable didactical tools to help the students with any concept abstraction difficulties, as they produce a playful environment to develop classes, stimulate cognitive processes like perception, memory, language, thought and permit the modeling of real events that evolve over time^(^ 11 ^)^.

### Improvements

*(...) I did not notice the arrows inside the text balloons sometimes, perhaps enlarging them would be ideal* (Part16); *(...) just some balloons that disappear after a while (...) the forward symbol is missing in the demonstration in figures of the procedure* (Part12); *(...) the animations are excellent, but I was unable to watch most of the videos again, as it "blocked" in the end* (Part13).

As perceived, adding themes interconnected with the procedure studied was suggested, as follows: *(...) highlighting the importance of using procedural gloves (IPE) in the medication administration, aiming for professional protection* (Part03); *(...) showing the hand washing scene (...)* (Part06).

These comments suggest the need to better advise the participants about how to use the learning object, as well as an adaptation in the elaboration and arrangement of the content, in order to prevent possible problems in handling and visualizing the material on the computer.

## Discussion

In nursing, the need for innovations in the teaching-learning process is observed, which stimulates the knowledge and autonomous and independent learning through the adoption of learning objects. It is evidenced that the use of computer technologies permits more creative teaching processes, furthering the students' more active learning^(^ 4 ^)^.

Studies show that young people increasingly show interest in technology, increasing the mean number of weekly accesses for educational purposes. These findings support the adoption of technology use in teaching to permit autonomous and interactive learning^(^ 12 ^-^ 14 ^)^.

The educational aspects of the LO assessed demonstrate that the theme is relevant for nursing and that the objectives and content are pertinent. Medication administration, a technique that is frequently used in health institutions and is apparently simple, should be a well-established practice, as an error can entail severe consequences. This is not unreal in health, as reports of injuries, tissue necrosis, muscle group contractions, fibroses and even loss of joint movement range were found in the literature with regard to children and adults who used intramuscular medication^(^ 3 ^)^.

There are themes related to intramuscular medication administration that were not focused on in this study, but which are nevertheless essential. The evaluators suggested the following, for example: use of IPE (Individual Protection Equipment) by professionals and the humanization process with the patient.

The LOA as computer technology is a resource that can be used to support the learning. Its main idea is to "break" the educational contents into small parts that can be reused in different learning environments. Any electronic material that contains information that contributes to the knowledge construction can be considered a learning object, whether in the form of an image, a *HyperText Markup Language* (HTML) page, an animation or simulation^(^ 1 ^)^.

The intramuscular medication administration procedure is complex and includes other techniques besides the puncture procedure itself, demanding flexible technological resources that can help to train the nursing professionals. The elaborated media dynamically and objectively explored medication administration themes. It should be highlighted that the LO can serve as a complementary resource for nursing undergraduates and professionals.

The presentation of hypertexts and images needs to comply with the criteria of visual appropriateness, being attractive and understandable for the students and allowing them to navigate as they desire, respecting their learning time^(^ 15 ^)^.

The fact that the LO is hosted in a VLE available on the Internet facilitates the network access and grants flexibility as to the time and place to study. This fact supports the autonomous and independent learning perspective and the establishment of the students' individual learning rhythms.

The evaluators also assessed the presentation of the LO's didactical resources as appropriate, which were planned in the design phase of the LO based on the educational objectives. Design is defined as the planning, development and systematic use of teaching methods, techniques and teaching activities for technology-supported educational projects. Furthermore, it is highlighted that this process is not limited that the visible part of instructional products, nor does it simply refer to abstract teaching planning, but it reflects the articulation between form and function, in order to comply with the proposed educational objectives^(^ 5 ^)^.

When using the web, actions like updates, storage and recovery, information distribution and instantaneous sharing become possible; as well as the overcoming of time and space limits; the construction of knowledge by the subject, of collaborative and cooperative learning, of the subjects' greater autonomy; the development of collective intelligence^(^ 16 ^)^.

These experiences with the use of VLE have demonstrated that the interactivity among the subjects is fundamental in the learning process, as these virtual communities are electronic networks of self-defined interactive communication, organized around an interest or goal that is shared by a group of individuals with common interests, who exchange experiences and information. The experiences with the use of VLE have demonstrated that the interactivity among the subjects is fundamental in the learning process^(^ 16 ^)^.

Based on the comments and suggestions, the contributions and limits of the LO could be highlighted, considering it as an innovative resource that can contribute to the teaching-learning process in nursing, indicating improvements and refinement needs of the media according to the needs of the target public it is to be applied to.

## Conclusion

This study was elaborated to develop a learning object about intramuscular medication administration and assess it from the perspective of nursing undergraduates and nursing professionals.

The object was developed in accordance with a planning that permitted the construction of a flexible, dynamic, clear, objective and easily understandable resource, addressing a relevant theme for nursing.

Most of the nurses (8) and nursing undergraduates (8) assessed the criteria related to the educational aspects, environment interface and didactic resources as excellent and satisfactory, resulting in 124 (97%) positive answers, so that the LO was considered appropriate for nursing teaching.

The evaluators' comments indicate that the educational technology has a clear language, objectives appropriate to the target public and easily understandable and appropriate texts and audiovisual resources. They also indicate that the teaching method could be applied to other themes, contributing to the education and training of nursing professionals.

The adoption of the learning object IM medication administration can positively influence nursing teaching, stimulating knowledge, autonomous and independent learning, aligned with the new professional education requirements.
